# SVUPP: Pre-phasing long reads improves structural variant genotyping

**DOI:** 10.1093/bioinformatics/btaf587

**Published:** 2025-10-24

**Authors:** Zilong Li, Frederik Filip Stæger, Robert W Davies, Ida Moltke, Anders Albrechtsen

**Affiliations:** Section for Computational and RNA Biology, University of Copenhagen, Copenhagen 2200, Denmark; Section for Computational and RNA Biology, University of Copenhagen, Copenhagen 2200, Denmark; Department of Statistics, University of Oxford, Oxford OX1 3LB, United Kingdom; Genomics plc, Oxford OX1 1JD, United Kingdom; Section for Computational and RNA Biology, University of Copenhagen, Copenhagen 2200, Denmark; Section for Computational and RNA Biology, University of Copenhagen, Copenhagen 2200, Denmark

## Abstract

**Summary:**

Here, we present an approach, called SVUPP, which improves genotyping of structural variant (SV) by incorporating read phasing information into genotype likelihoods. Through comprehensive benchmarking, we show that SVUPP achieved higher accuracy than cuteSV2, Sniffles2 and kanpig with both long and ultra long Oxford Nanopore Technologies (ONT) data as well as Pacific Biosciences (PacBio) HiFi data for genotyping SVs without close neighbor SVs. SVUPP can be applied together with SV callers such as cuteSV2 and take the per-read phasing information from reference panel based phasing method such as QUILT2 or from reference-free phasing method such as WhatsHap.

**Availability and implementation:**

SVUPP is written in Nextflow with modular design and is freely available here https://github.com/Zilong-Li/SVUPP.

## 1 Introduction

Structural variants (SVs) are typically defined as genetic variants that are larger than 50 base pairs, and include deletions, duplications, inversions, translocations, and more complex rearrangements (Collins *et al.* 2020). By virtue of their size, SVs can affect larger regions of the genome, potentially disrupting gene function, altering regulatory elements, or creating novel gene fusions (Hurles *et al.* 2008) and are important contributors to genetic diversity, evolution, and disease (Talkowski *et al.* 2012, Weischenfeldt *et al.* 2013). Thus, accurate detection and characterization of SVs is crucial. However, genotyping SVs presents significant challenges, even with high-throughput long read sequencing technologies. In fact, state-of-the-art SV genotyping methods such as kanpig (English *et al.* 2025), Sniffles2 (Smolka *et al.* 2024), and cuteSV2 (Jiang *et al.* 2025) all show a substantial amount of genotyping error even with high quality > 30X Pacific Biosciences (PacBio) HiFi and Oxford Nanopore Technologies (ONT) R10 data (English *et al.* 2025).

In this work, we describe a new approach to genotyping SVs, SVUPP, which integrates reads phasing information into genotype likelihoods in order to improve genotype calls. Using the recent benchmarking truth set from Platinum pedigree (Kronenberg *et al.* 2025, Porubsky *et al.* 2025), we show that SVUPP has lower genotype discordance rate than kanpig, cuteSV2, and Sniffles2 with both ONT and PacBio HiFi data. Additionally, we demonstrate that the use of SVUPP leads to lower Mendelian error rate when applying common SV discovery pipelines to ONT Simplex data at 10X with six trios from the 1000 Genomes Project.

## 2 Methods

Inspired by methods for short sequencing reads (DePristo *et al.* 2011, Korneliussen *et al.* 2014), we use genotype likelihoods (GL) to incorporate the uncertainty of the data. Specifically, for incorporating phasing information into the SV genotype likelihoods, we code genotypes as ordered pairs of alleles, using 0 and 1 for the reference and alternative allele respectively. We denote the phased diploid genotype as $H=(H_{1},H_{2})$ and $H_{h}\in \{0,1\}$, where *h* arbitrarily represents maternal or paternal haplotype. For an SV in the individual, let *D* be the number of reads covering the SV and let *r* be the read index. Then we derive the phased genotype likelihood as


$$P(X|H)\propto \prod_{r=1}^{D}\sum_{h\in \{1,2\}}P(X_{r}|H_{h})P({\text{hap}}_{r}=h),$$

where *D* represents the number of reads covering the position, $X_{r}\in \{0,1\}$ indicates whether the SV allele on read *r* is the reference or the alternative allele, and $P(hap_{r}=h)$ is the probability of read *r* belonging to haplotype *h*. The SV assignment for the reads are performed using cuteSV2 and can sometimes be wrong. Therefore, we introduce an SV assignment error term, e=0.01. The probability of observing a certain SV allele on read *r* from a certain haplotype can be written as $P(X_{r}|H_{h})=1-e$ if $X_{r}=H_{h}$ or $P(X_{r}|H_{h})=e$ if $X_{r}\neq H_{h}$. Therefore, we can derive the GL from the phased GL. Let G∈{0,1,2} be the diploid genotype, which counts the number of alternative alleles. We have


$$P(X|G)=\{\begin{array}{cc} P(X|H=(0,0)) & \;\text{if}\;G=0\\ \frac{1}{2}P(X|H=(0,1))+\frac{1}{2}P(X|H=(1,0)) & \;\text{if}\;G=1\\ P(X|H=(1,1)) & \;\text{if}\;G=2 \end{array}.$$

We call genotypes by choosing the genotype that maximizes the genotype likelihoods. We calculate posterior genotype probability (GP) of each genotype assuming a uniform prior by


$$GP_{g}=P(G=g|X)=\frac{P(X|G=g)}{\sum_{g^{\prime }=0}^{2}P\left(X|G=g^{\prime }\right)}.$$

Then, we derive genotype quality (GQ) as the Phred scale of GP for the most probable genotype, i.e. GQ=−10 log 10(1−max(GP)).

### 2.1 Measuring genotype discordance

First, we identified the SVs with close neighbor SVs within 1000 bases in the truth set using the command “truvari anno numneigh -r 1000” (English *et al.* 2022), and divided the SV list into two. Then, for each SV category, we used vcfppR (Li 2024) to extract genotype calls (GT) and genotype quality (GQ) from the output VCF of each genotyping tool. We calculated genotype discordance for all GQ thresholds in R (R Core Team 2023) as the proportion of GT that were not identical to the autosomal GT in the truth set. For example, at a GQ-threshold of 30, we included all genotype calls with a GQ≥30, see Fig. 3, available as supplementary data at *Bioinformatics* online.

**Figure 1.**
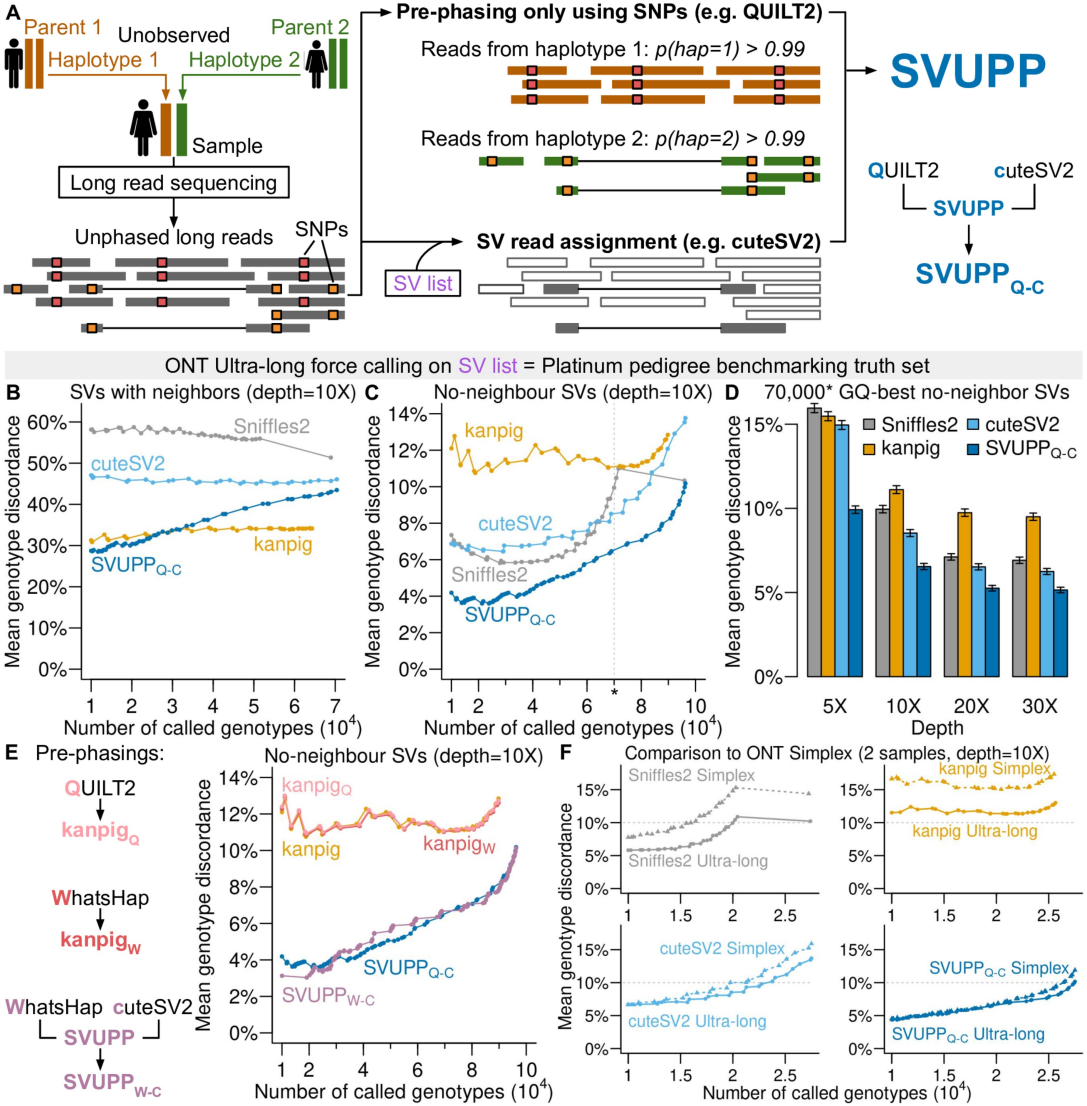
Benchmarking of genotyping accuracy against Platinum Pedigree truth set. (A) SVUPP method overview. Note that reads with haplotype probability <0.99 are still used. (B) Genotype discordance rates as a function of the number of genotypes called from ONT ultra-long read data downsampled at 10X, including only on SVs with at least 1 neighbor SVs within 1000 bp. The genotypes are sorted by genotype quality (GQ) across all individuals, so e.g. a point at 70 000 on the *x*-axis shows the mean discordance of the 70 000 genotypes with highest GQ. (C) As (B) but including only SVs with no neighbor SVs within 1000 bp. (D) Mean genotype discordance for the 70 000 genotypes with highest QC based on data downsampled to depths 5X, 10X, 20X, and 30X. (E) Mean genotype discordance for SVUPP and kanpig each combined with different read pre-phasing strategies. (F) Mean genotype discordance for all genotypes applied to two types of data; ONT Ultra long read data and ONT Simplex data both with 10X depth.

To compare fairly across depths, we used the same number of genotype calls across all depths and methods. For example, we chose the 70 000 best genotype calls ranked by GQ in Fig. 1D since this is the approximate number of genotype calls with non-zero GQ from Sniffles2. Using a specific number of genotype calls meant that it did not correspond exactly to a GQ-threshold and we had to randomly choose genotype calls within the last GQ bin.

### 2.2 Measuring Mendelian patterns and errors

For each SV, genotype patterns were created within each trio as the genotype of one parent, the genotype of the second parent, and the genotype of the child; e.g. ‘012’, means parent one has genotype 0 = 0/0, parent two has genotype 1 = 1/0, and the child has genotype 2 = 1/1. This results in 27 possible Mendelian patterns, but can be simplified to 10 patterns by ignoring parent order and polarizing to the minor allele within the trio (Table 3, available as supplementary data at *Bioinformatics* online). Each Mendelian pattern was ranked by the minimum GQ within the trio.

## 3 Results

### 3.1 Method overview

We propose an approach for genotyping SVs from long read sequencing data, which adds read phasing information into existing methods, as depicted in Fig. 1A. First, long reads from the target individual are pre-phased using only single nucleotide polymorphism (SNP) information and no structural variant (SV) information, i.e. each read is probabilistically assigned to the individual’s two parental haplotypes. Second, given an SV list each read is assigned as either carrying a given SV allele or matching the reference. Finally, the parental haplotype probability and the read assignment are used to calculate SV genotype likelihoods (GLs), which form the basis for genotype calling and genotype quality (GQ) as described in the Methods section. For examples that illustrate why pre-phasing of reads can lead to improved genotype calling, see Fig. 1, available as supplementary data at *Bioinformatics* online.

In most analyses, we performed the read pre-phasing step with QUILT2 (v2.0.4) (Li *et al.* 2024), which uses the large UK biobank haplotype reference panel (Bycroft *et al.* 2018). For the SV read assignment step, we used cuteSV2. But we replaced the genotyping module in cuteSV2 with our genotyping formula. We note that other tools than QUILT2 and cuteSV2 can be used; the proposed approach is to incorporate SNP based read phasing information into the GL calculation. We call this approach **S**tructural **V**ariant genotype calling **U**sing **P**re-**P**hased reads (SVUPP) and when used in combination with QUILT2 and cuteSV2, we refer to it as SVUPP${}_{Q-C}$.

### 3.2 Improved genotype calling on Platinum Pedigree ONT data

To assess the performance, we applied SVUPP${}_{Q-C}$ and the commonly used SV genotyping methods cuteSV2 (v2.1.1), Sniffles2 (v2.3.3) and kanpig (v1.0.2) to the Platinum Pedigree ONT ultra-long read (mean read length = 72 kb) data (Kronenberg *et al.* 2025). We used the 7 individuals from the second and third generation in the pedigree that is published along with high quality SV genotypes and SV list, which we used as a truth set. We used forced calling, meaning we forced all the methods to call genotypes for the exact same set of SVs, namely those that are included in the truth set. For assessing the effect of sequencing depth we downsampled the data to different depths. We used 10X for most of our benchmarking, since this mimics currently generated data from the 1000 Genomes Project. Moreover, we note that genotype quality (GQ) is an important metric that has been used in benchmarking (Saunders *et al.* 2025) and empirical data analyses (Kronenberg *et al.* 2025, Schloissnig *et al.* 2025). Thus, we will stratify the results by GQ, which implicitly reflects genotype call rates.

First, we analyzed ultra long ONT data. As in English *et al.* 2025, we divided the SVs into two categories: SVs with neighbors (at least one other SV within 1000 bases) and no-neighbor SVs, due to their difference in accuracy. For the SVs with close neighbors, kanpig had the lowest mean genotype discordance (Fig. 1B). However, none of the methods performed well. In fact, the mean genotyping discordance for all methods was >30% regardless of how strictly we filtered for genotype quality (GQ). Due to the very low accuracy across all methods, we will not focus on this category of SVs in the rest of this article. Furthermore, we found that in the benchmarking there is a large jump in accuracy for Sniffels2, which is due to many SVs having the lowest possible GQ=0 when there are no supporting reads.

**Figure 2.**
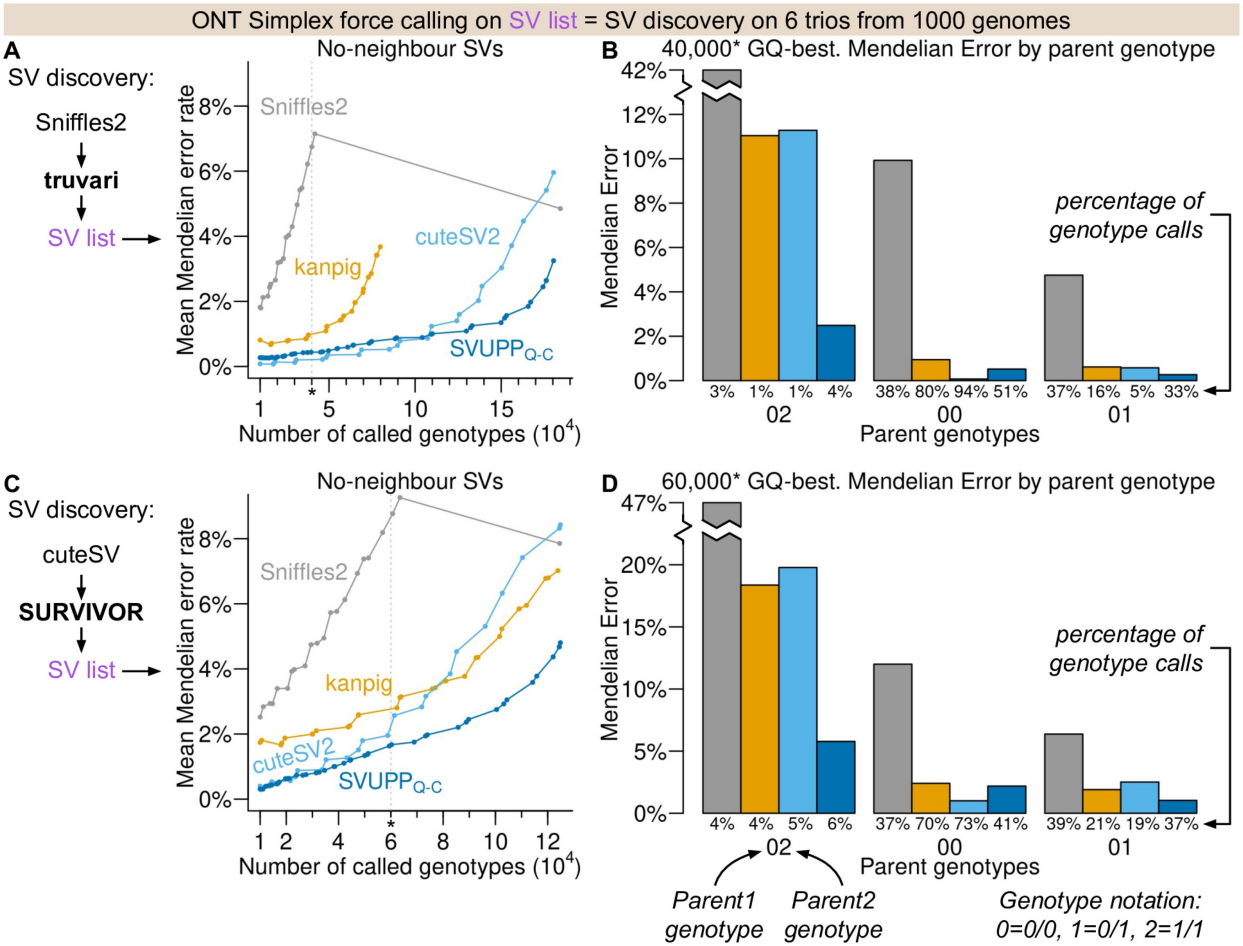
Mendelian errors in analyses of six trios for inferred SV lists. (A) Mean Mendelian error rate for each method when genotyping the SVs discovered by a pipeline that uses Sniffles2 and Truvari. (B) Mendelian error rates stratified by parents’ genotype patterns for the 40 000 GQ-best called genotypes complementing results in (A). We chose 40 000 based on the approximate number of genotypes for which Sniffles2 has GQ > 0. (C) As (A) but based on an SV list inferred using a pipeline that uses cuteSV2 and SURVIVOR. (D) Same as (B) but based on an SV list inferred using cuteSV2 and SURVIVOR and for the 60 000 genotypes with highest GQ. We chose 60 000 based on the approximate number of genotypes for which Sniffles2 has GQ > 0.

Around 60% of SVs had no neighbors and had a markedly lower genotype discordance rate, with SVUPP${}_{Q-C}$ performing better than all the other methods for any number of genotypes included (Fig. 1C). Without a GQ filter, both cuteSV2 and SVUPP had a 100% call rate, whereas Sniffles2 and kanpig call fewer genotypes corresponding to a call rate of 99.7% and 91.4%, respectively (Table 1, available as supplementary data at *Bioinformatics* online). However, for SV genotype calls with GQ>0, Sniffles2 had the lowest call rate of 74.4%, corresponding to around 70 000 SV genotype calls. In order to make a fair comparison, we selected the 70 000 SV genotype calls with the highest GQ from each method and found genotype discordance rates of 6.5% for SVUPP${}_{Q-C}$, 10.0% for Sniffles2, 8.5% for cuteSV2, and 11.1% for kanpig (Table 1, available as supplementary data at *Bioinformatics* online). The advantage of SVUPP${}_{Q-C}$ increases as the GQ threshold becomes stricter. Interestingly, the improvement was particularly pronounced for insertions, where all four methods had higher genotype discordance rates than for deletions, especially Sniffles2 and kanpig (Fig. 2, available as supplementary data at *Bioinformatics* online).

Next, we assessed the performance at different mean sequencing depths: 5X, 10X, 20X, and 30X. Not surprisingly, the discordance rates for all four methods were higher for lower depths (Fig. 1D, Table 2, available as supplementary data at *Bioinformatics* online). However, the discordance rates improved little for 30X compared to 20X. Nevertheless, SVUPP${}_{Q-C}$ performed better for all GQ thresholds across all the tested depths (Fig. 3, available as supplementary data at *Bioinformatics* online).

### 3.3 No effect of phasing software choice

Next, we tried to assess whether using the pre-phasing also improves kanpig, which can take haplotype tagging information as input. We also tested whether we could use another phasing tool, WhatsHap, which is not dependent on the SNP reference panel, but instead relies on phased SNP genotypes of target individuals from e.g. high coverage short reads sequencing (Patterson *et al.* 2015). Interestingly, the mean genotype discordance for kanpig did not improve after being provided haplotype tagging information, either from QUILT2 (v2.0.4) or WhatsHap (v1.7). Furthermore, SVUPP performed similarly with WhatsHap and QUILT2 (Fig. 1E), which means that SVUPP can be used for non-model organisms, where a reference panel is not available.

### 3.4 SVUPP also improves performance on ONT Simplex and PacBio HiFI data

To evaluate the performance on the commonly used shorter ONT Simplex long read data, we analyzed the two Platinum Pedigree project individuals, NA12877 and NA12878, who were also sequenced with ONT Simplex (mean read length = 12 kb) as part of the 1000 Genomes Project. We found that SVUPP${}_{Q-C}$ was insensitive to the type of ONT data. In contrast, Sniffles2 and kanpig performed much worse for the shorter simplex reads compared to ultra long reads as shown in Fig. 1F. Importantly, SVUPP${}_{Q-C}$ showed a much lower genotype discordance compared to the other methods on ONT Simplex data (Fig. 4, available as supplementary data at *Bioinformatics* online). In addition, with the more accurate PacBio HiFi reads, all methods showed improved accuracy compared to ONT data with kanpig showing the most pronounced improvement (Fig. 5, available as supplementary data at *Bioinformatics* online). Nevertheless, SVUPP${}_{Q-C}$ still performed the best for genotyping SVs with no close neighbors.

### 3.5 Improved genotype calling when a list of structural variants is not available

Unlike for the Platinum Pedigree dataset, a high quality SV list and genotype truth set is not available for most real datasets. Thus, to assess the performance on other datasets, we had to rely on SV discovery pipelines and Mendelian errors as a proxy for genotype discordance. We therefore assessed performance using the 6 trios from the 1000 Genomes Project with ONT Simplex data. We inferred the SV list using two common SV discovery pipelines: Sniffles2 (v2.3.3) combined with Truvari (v4.2.2) (English *et al.* 2022) and cuteSV2 (v2.1.1) combined with SURVIVOR (v1.0.7) (Jeffares *et al.* 2017). It should be noted that Mendelian errors will not capture all genotype errors (Fig. 6, available as supplementary data at *Bioinformatics* online), e.g. if both parents are heterozygous, then no genotype in the child will result in Mendelian errors, so genotype errors in the child will not be captured. However, for the Platinum Pedigree truth set the overall difference in genotyping error between methods is also reflected in the Mendelian errors (Fig. 6A, available as supplementary data at *Bioinformatics* online).

In general, we found that Sniffles2 combined with Truvari inferred more SVs and genotyping these SVs led to lower Mendelian errors compared to inferring SVs with cuteSV2 combined with SURVIVOR (Fig. 2A and C and Figs 7 and 8, available as supplementary data at *Bioinformatics* online). For both SV lists, SVUPP${}_{Q-C}$ had lower overall Mendelian error rates than both Sniffles2 and kanpig across all GQ thresholds (Fig. 2A and C). SVUPP${}_{Q-C}$ also had lower Mendelians errors than cuteSV2 when all genotypes were considered, but cuteSV2 performed slightly better for the more stringent GQ in the SV list inferred by Sniffles2 and Tuvari (Fig. 2A). However, when stratifying by the three parent genotype combinations, we found that kanpig and cuteSV2 had a much higher percentage of genotype calls (>70%) where both parents are homozygous for the same allele (Fig. 2B and D). This category contains many hidden genotype errors not captured with Mendelian errors (Fig. 6D, available as supplementary data at *Bioinformatics* online) suggesting that we are underestimating the genotype error rate, particularly for cuteSV2 and kanpig. Notably SVUPP${}_{Q-C}$ had a much lower error rate than the other methods for the sites where the parents are called as discordant homozygous (Parent genotype 02). At these sites, any error in the child will lead to a Mendelian error.

## 4 Discussion

We presented SVUPP, an approach that improves SV genotyping accuracy in existing methods by integrating the per-read phasing information into genotype likelihoods. Compared to other state-of-the-art SV genotyping methods, where mainly allele depth at a single locus is considered, SVUPP additionally leverages the phasing information from adjacent SNPs.

The benefit of SVUPP was very clear for SVs that do not have other close SVs across all tested depths. For complex regions with multiple SVs, where SV calling is challenging (Qin and Li 2025), all methods had very high discordance rates, but kanpig performed relatively better. Both Sniffles2 and kanpig performed much worse on the shorter ONT Simplex reads than on ONT Ultra-long reads, while cuteSV2 and SVUPP worked well on both, with SVUPP being the best. Interestingly, all methods perform worse on insertions than on deletions, especially Sniffles2 and kanpig. We show that considering the number of genotype calls ranked by genotype quality is essential for fair benchmarking between methods. On computational performance, all methods showed a low memory footprint. The reads phasing step dominates the runtime of SVUPP while in the SV genotyping step SVUPP${}_{Q-C}$ showed similar speed to others (Fig. 9, available as supplementary data at *Bioinformatics* online).

This study was limited by the low number of publicly available SV genotype truth sets, which makes benchmarking challenging, since Mendelian error is only a proxy for genotype discordance. In addition, when performing both SV calling and genotype calling, we need to merge the SVs between individuals and from the Mendelian errors we cannot evaluate which errors come from the SV merging and which are from the SV genotyping. However, based on both the benchmarking with the trust set and the benchmarking with Mendelian errors, it was clear that pre-phasing of reads improves genotyping of SVs markedly. Finally, SVUPP currently assumes a uniform prior, which assigns equal prior probability to each genotype, which is the same as cuteSV2 but in contrast to others, e.g. Sawfish (Saunders *et al.* 2025). Hence, further study of different priors could be explored to increase performance. Especially, there is a great potential in using haplotype imputation methods to refine the genotypes by modeling local linkage disequilibrium patterns and haplotype sharing information in the population (Browning and Browning 2007, Davies *et al.* 2016). Further methodological development can benefit from modeling linkage disequilibrium and SNP-SV physical linkage in a multiple-sample context.

## Supplementary Material

btaf587_Supplementary_Data

## Data Availability

The source code of SVUPP is publicly available at Github: https://github.com/Zilong-Li/SVUPP. All code, scripts and assessment results are provided as a Zenodo dataset at https://doi.org/10.5281/zenodo.17227287. The sequencing data and benchmark datasets are publicly available as detailed in Supplementary Notes.
