# Supplementary material for: SVUPP: Pre-phasing long reads improves structural variant genotyping
Source: Bioinformatics. 2025 Oct 24;41(12):btaf587. doi: 10.1093/bioinformatics/btaf587 (PMC12771361; doi:10.1093/bioinformatics/btaf587)
Supplement: btaf587_Supplementary_Data [file btaf587_Supplementary_Data.pdf]

Supplementary of “Pre-phasing long reads improves structural variant genotyping”

Supplementary Notes

Datasets for benchmarking

For evaluation of genotype concordance, we use the pedigree-validated truth set of SVs made for benchmarking purposes [1]. We only downloaded the BAM files aligned to hg38 reference and the VCF files with true genotypes for 7 individuals in the pedigree, who consent to make it public(https://github.com/Platinum-Pedigree-Consortium/Platinum-Pedigree-Datasets). For evaluation of Mendelian errors using ONT simplex data, we downloaded the recently aligned CRAM files for 6 trios from the 1000 Genomes Project [2]. However, these published CRAM files from https://ftp.1000genomes.ebi.ac.uk/vol1/ftp/data_collections/1KG_ONT_VIENNA/ did not store the base qualities of each read, i.e. the QUAL field. Therefore, we first extracted the raw reads from CRAM and converted them into FASTQ files. Then we run *minimap2*[3] with options *‘-y -t 28 -ax map-ont -R’* to realign them against the hg38 reference.

Software and command lines

We run all SV calling and genotyping tools with default parameters or the suggested preset parameters according to different types of long read data. In detail, the command line of Sniffles (v2.3.3) and kanpig (v1.0.2) for both ONT and PacBio HiFi data are the same, which are as follows:

sniffles --input {bam} --genotype-vcf {sv.vcf} --vcf {id}.vcf --tandem-repeats {bed}
kanpig gt --input {sv.vcf} --reads {bam} --reference {fasta} --out {id}.vcf --sample {id}

The command line of cuteSV (v2.1.1) and the forked version of cuteSV (https://github.com/Zilong-Li/cuteSV v0.0.2 was used in SVUPP) for ONT data are the same, which is:

cuteSV {bam} {fasta} {out.vcf} {outdir} -Ivcf {sv.vcf} --max_cluster_bias_INS 100 --diff_ratio_merging_INS 0.3 --max_cluster_bias_DEL 100 --diff_ratio_merging_DEL 0.3 --min_support 1

The command-line of cuteSV (v2.1.1) and the forked version of cuteSV (Zilong-Li/cuteSV v0.0.2 was used in SVUPP) for PacBio HiFi data are the same, which is:

cuteSV {bam} {fasta} {out.vcf} {outdir} -Ivcf {sv.vcf} --max_cluster_bias_INS 1000 --diff_ratio_merging_INS 0.9 --max_cluster_bias_DEL 1000 --diff_ratio_merging_DEL 0.5 --min_support 1

For the long reads phasing, QUILT (v2.0.4) and WhatsHap (v1.7) were used. Specifically, QUILT2 worked with a large reference panel from the UK Biobank 200K WGS data.

QUILT.R --prepared_reference_filename={ukb.panel.RData} --method=diploid --bamlist={bamlist} --bqFilter=20 --impute_rare_common=FALSE --downsampleToCov=60 --maxDifferenceBetweenReads=1e6 --use_mspbwt=TRUE --mspbwtL=5 --mspbwtM=1 --Ksubset=800 --Knew=800 --nGen=100 --buffer=500000 --chr={CHR} --regionStart={OREGS} --regionEnd={OREGE} --output_filename={out.vcf} --output_RData_filename={out.RData} --output_read_label_prob=TRUE

And WhatsHap employs two subcommands to haplotag the BAM with known SNP genotypes in a VCF.

whatshap phase {snps.vcf} {bam} --reference={fasta} -o {phased.snp.vcf}
whatshap haplotag {phased.snp.vcf} {bam} --reference {fasta}-o {tagged.bam}

In the above command lines, {bam} is the placeholder for the input bam file. {sv.vcf} and {snps.vcf} is the input VCF with known SVs and SNPs respectively, which are publicly available https://github.com/Platinum-Pedigree-Consortium/Platinum-Pedigree-Datasets. {fasta} is the GRCh38 reference used in long-read data alignment, which is available here https://storage.googleapis.com/brain-genomics-public/research/platinum/reference/GRCh38.no_alt_analysis_set.fa.gz. {bed} is a BED file used only by Snillfes that stores the region for tandem repeats, which is downloaded from the Github repository of Sniffles https://github.com/fritzsedlazeck/Sniffles/blob/master/annotations/human_GRCh38_no_alt_analysis_set.trf.bed.

References

1. Kronenberg, Z. *et al.* The Platinum Pedigree: a long-read benchmark for genetic variants. *Nature Methods* **22**, 1669–1676 (2025).

2. Schloissnig, S. *et al.* Structural variation in 1,019 diverse humans based on long-read sequencing. *Nature* **644**, 442–452 (2025).

3. Li, H. Minimap2: pairwise alignment for nucleotide sequences. *Bioinformatics* **34**, 3094–3100 (2018).

Supplementary Figures

Supplementary Figure 1

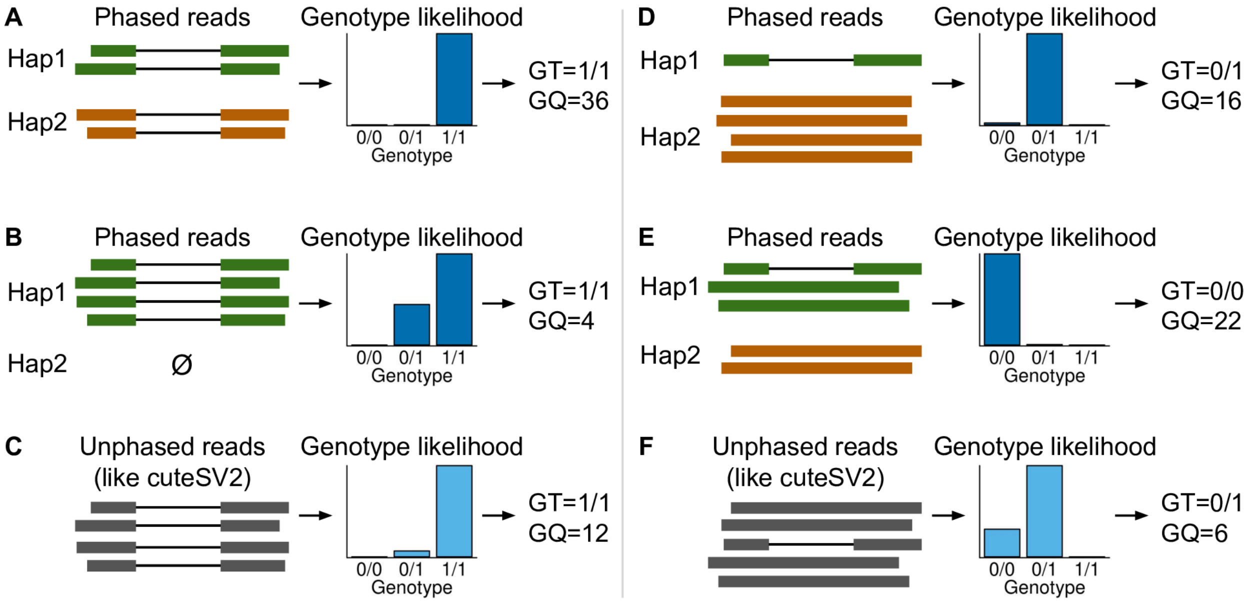

Supplementary Figure 1. Examples on how pre-phasing of reads changes the genotype calling and quality. In **A**, **B**, and **C**, four long reads contain the same deletion and the three examples show the effect of different pre-phasing results. In **A**, two reads are phased to each haplotype resulting in a high-quality 1/1 genotype, in **B** all the reads are from the same haplotype, which results in a low-quality 1/1 genotype, and in **C** the reads are unphased, which results in a 1/1 genotype with intermediate quality. In D, E, and F, one read contains a deletion and four reads do not, i.e. reference reads. In **D**, the deletion is from haplotype 1 and the four reference reads are from haplotype 2, which results in a 0/1 genotype with intermediate quality. In **E**, the deletion and two reference reads are from haplotype 1 and two reference reads are from haplotype 2 which results in a 0/0 genotype with slightly higher quality. In **F**, the reads are unphased which results in a 0/1 genotype with low quality.

Supplementary Figure 2

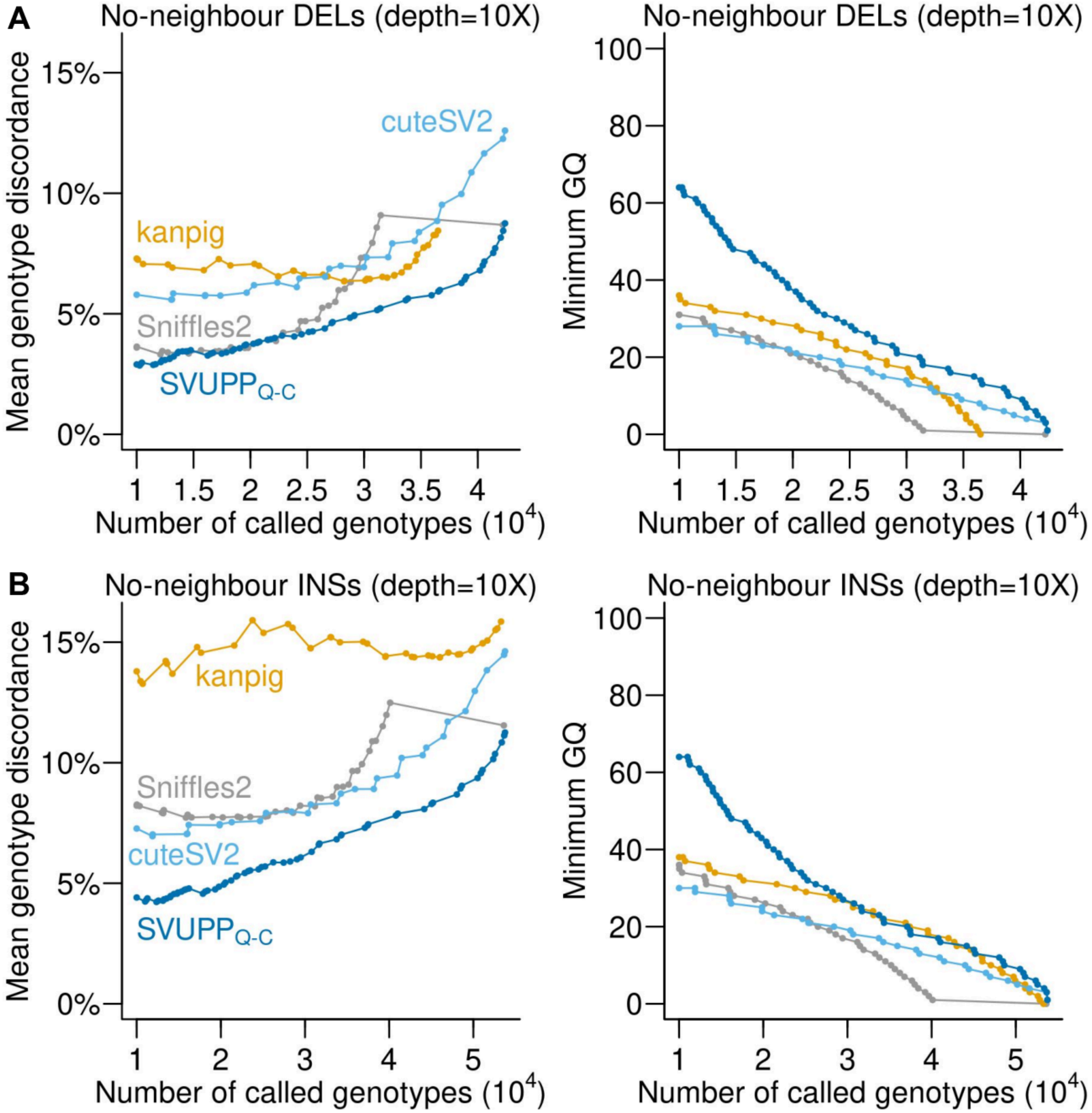

Supplementary Figure 2. Mean genotype discordance of no-neighbor SVs as in Figure 1C but split into **A.** deletions and **B.** insertions. The right plot shows the minimum genotype quality (GQ) as a function of the number of called genotypes. SVs in the Platinum pedigree benchmarking truth set were used for the evaluation.

Supplementary Figure 3

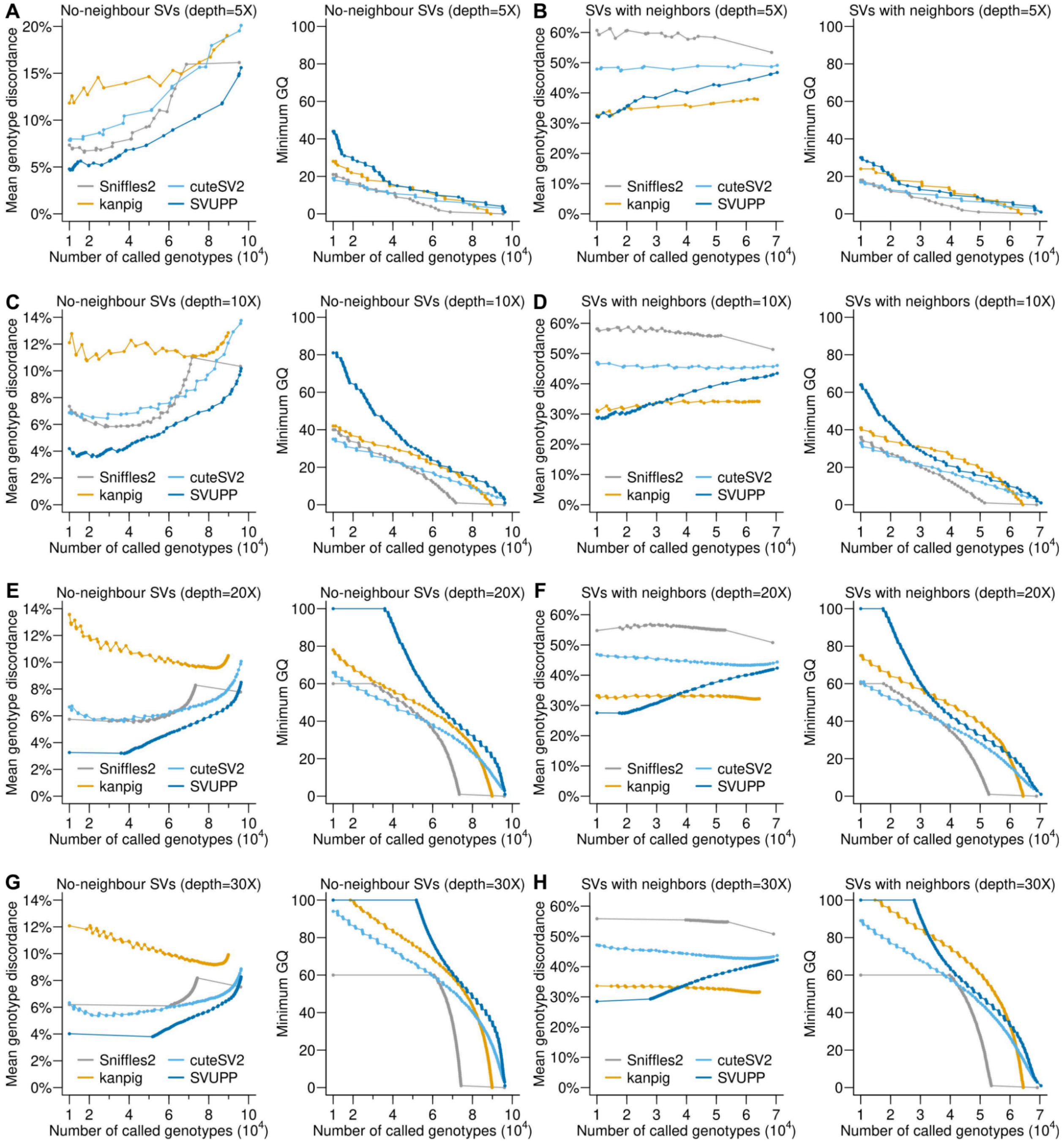

Supplementary Figure 3. **A, C, E, G.** Mean genotype discordance of no-neighbor SVs at different depths. **B, D, F, H.** Mean genotype discordance of SVs with neighbors at different depths. The right plot in each panel shows the minimum genotype quality (GQ) as a function of the number of called genotypes. SVs in the Platinum pedigree benchmarking truth set were used for evaluation.

Supplementary Figure 4

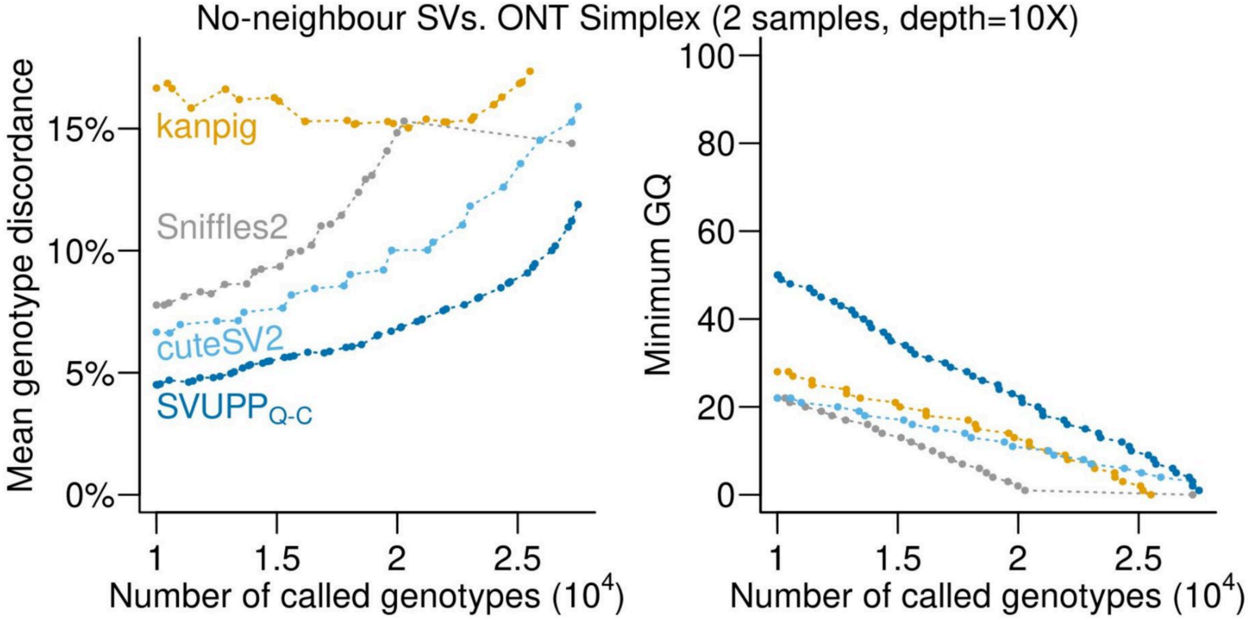

Supplementary Figure 4. Mean genotype discordance of no-neighbor SVs as in Figure 1F but summarizing all methods for ONT Simplex in one plot. The right plot shows the minimum genotype quality (GQ) as a function of the number of called genotypes. SVs in the Platinum pedigree benchmarking truth set were used for evaluation.

Supplementary Figure 5

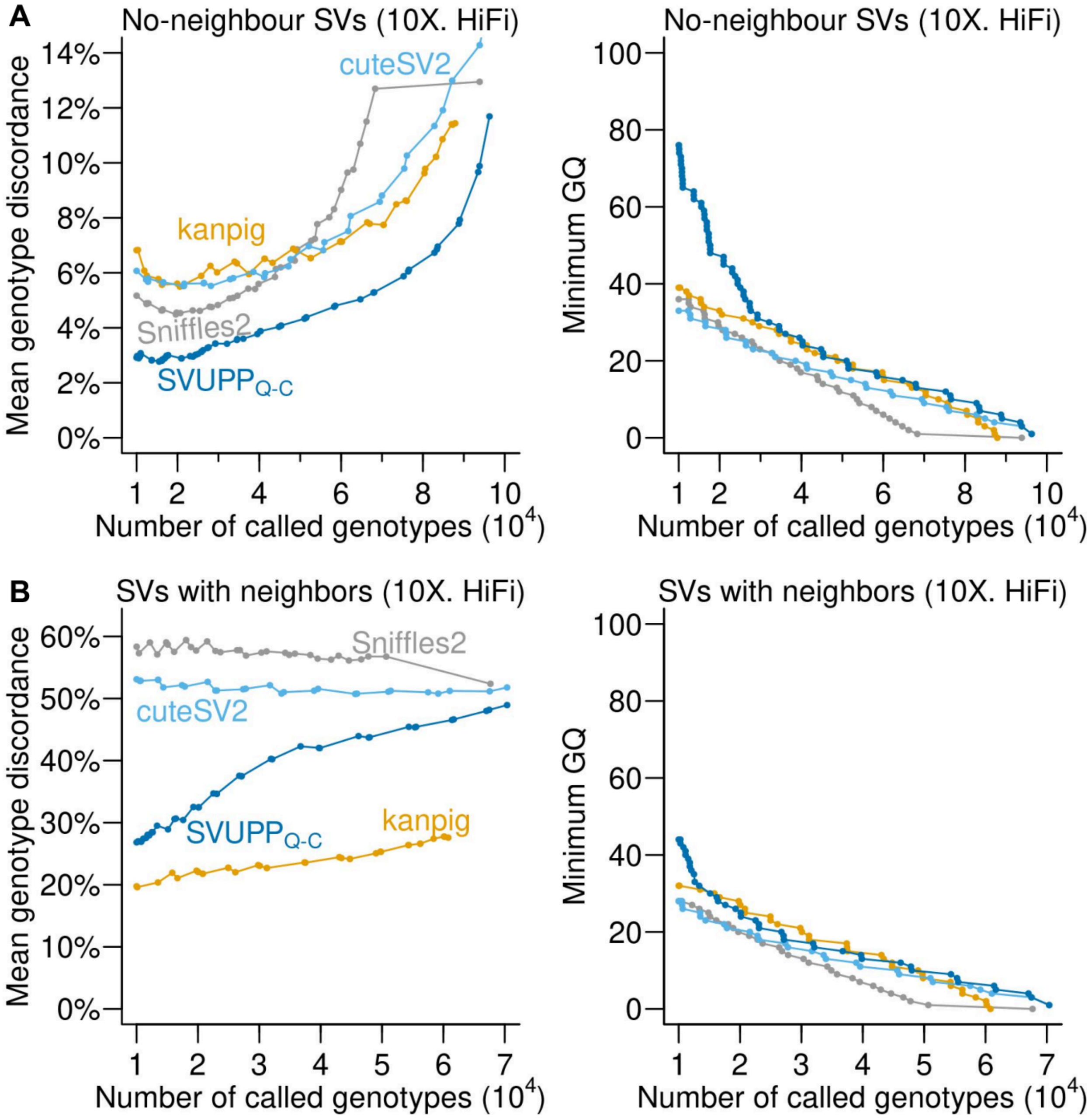

Supplementary Figure 5. Mean genotype discordance of no-neighbor SVs for 7 individuals as in Figure 1B,C but for PacBio HiFi data. The right plot shows the minimum genotype quality (GQ) as a function of the number of called genotypes. SVs in the Platinum pedigree benchmarking truth set were used for evaluation.

Supplementary Figure 6

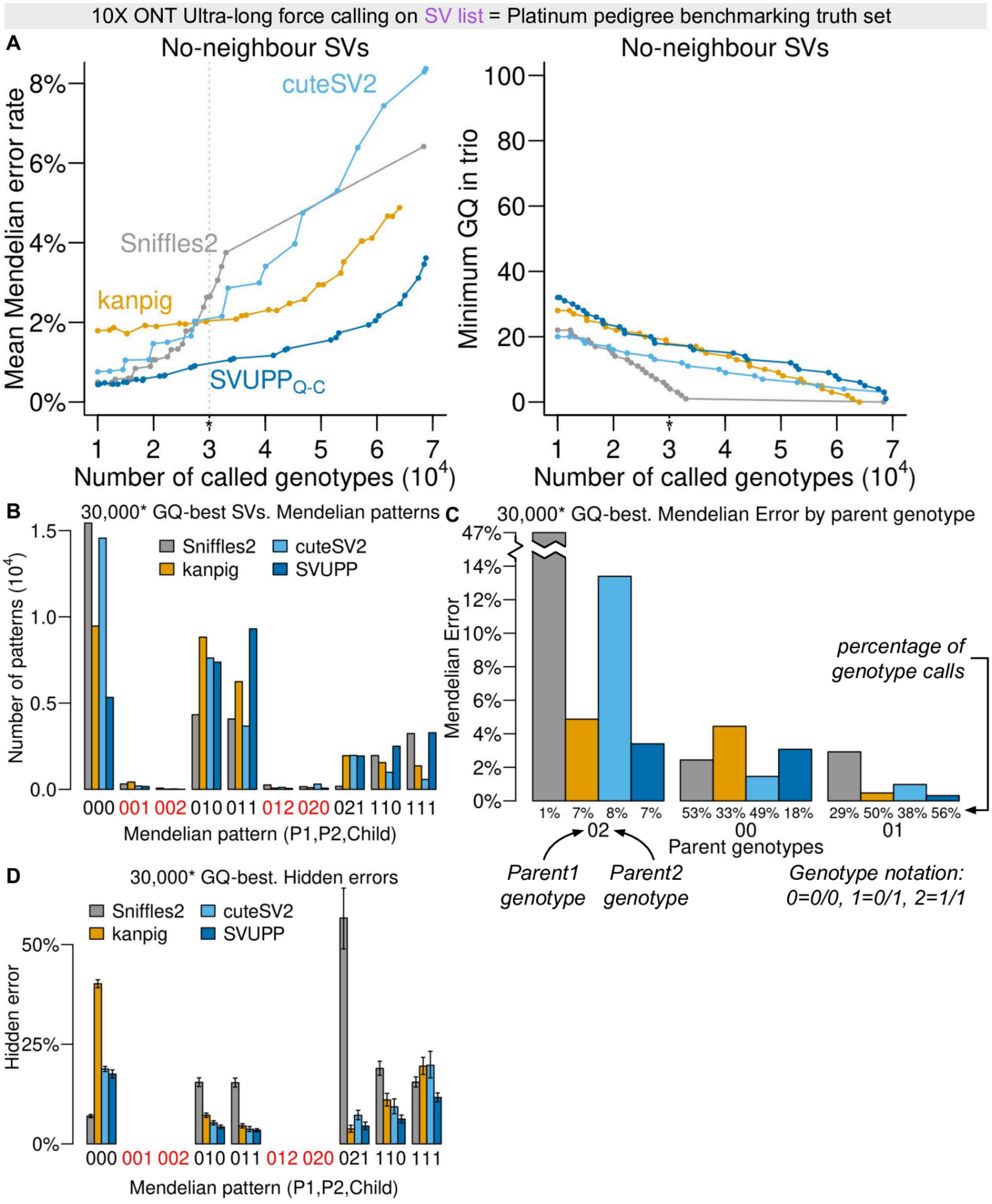

Supplementary Figure 6. Mendelian errors and patterns for No-neighbor SV for the Platinum pedigree with 10X ONT Ultra-long data. **A.** Mean Mendelian error rate as a function of the number of genotypes called (left) and minimum genotype quality (GQ) as a function of the

number of called genotypes to illustrate the connection between GQ threshold and number of genotypes called (left). **B.** Number of each of the different possible Mendelian patterns for each of the methods when considering the 30,000 GQ-best genotypes. **C.** Mendelian error rates stratified by parents' genotype patterns for the 30,000 GQ-best called genotypes complementing results in **A. D.** Proportion of hidden errors for each of the possible Mendelian patterns, with hidden errors defined as called genotypes that are discordant with the genotype in the truth set, but does not lead to a Mendelian error.

Supplementary Figure 7

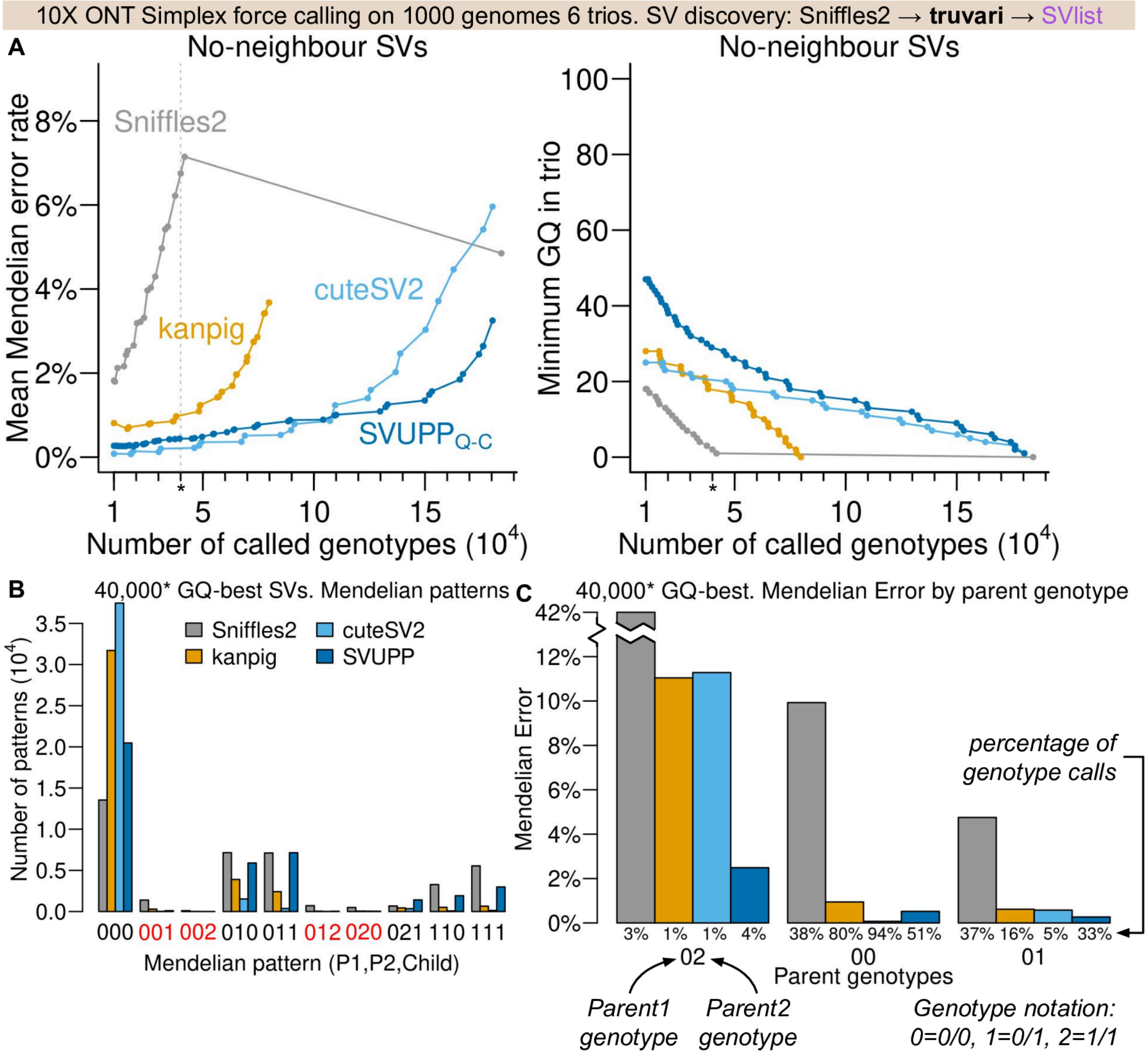

Supplementary Figure 7. Mendelian errors and patterns for re-genotyping 6 trios in 1000 genomes with SVs discovered by applying Sniffles2 and Truvari pipeline to 10X ONT Simplex data. **A.** and **C.** are exactly the same as Fig. 2 A,B.

Supplementary Figure 8

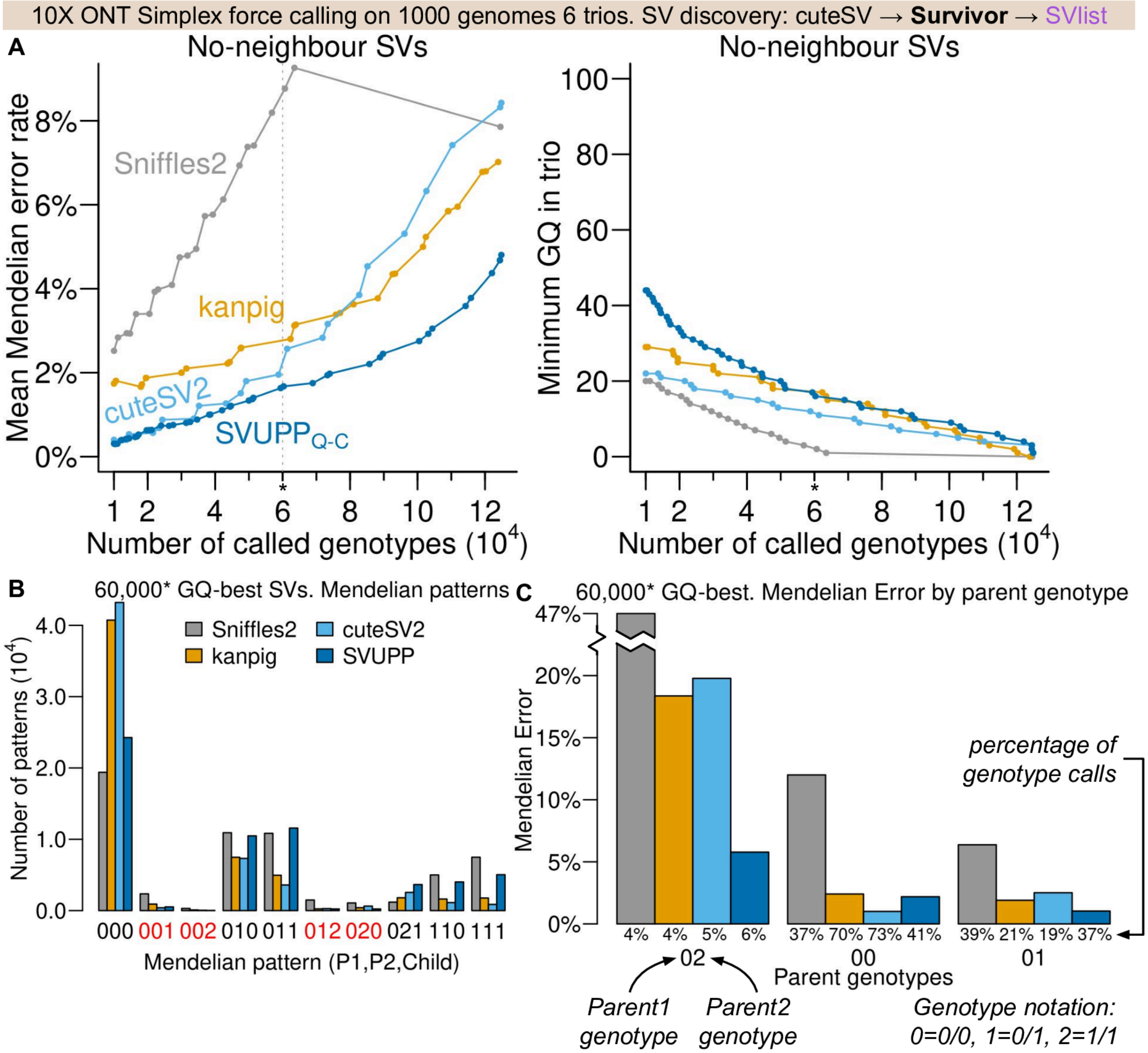

Supplementary Figure 8. Mendelian errors and patterns for re-genotyping 6 trios in 1000 genomes with SVs discovered by applying cuteSV and Survivor pipeline to 10X ONT Simplex data. **A.** and **C.** are exactly the same as Fig. 2 C,D.

Supplementary Figure 9

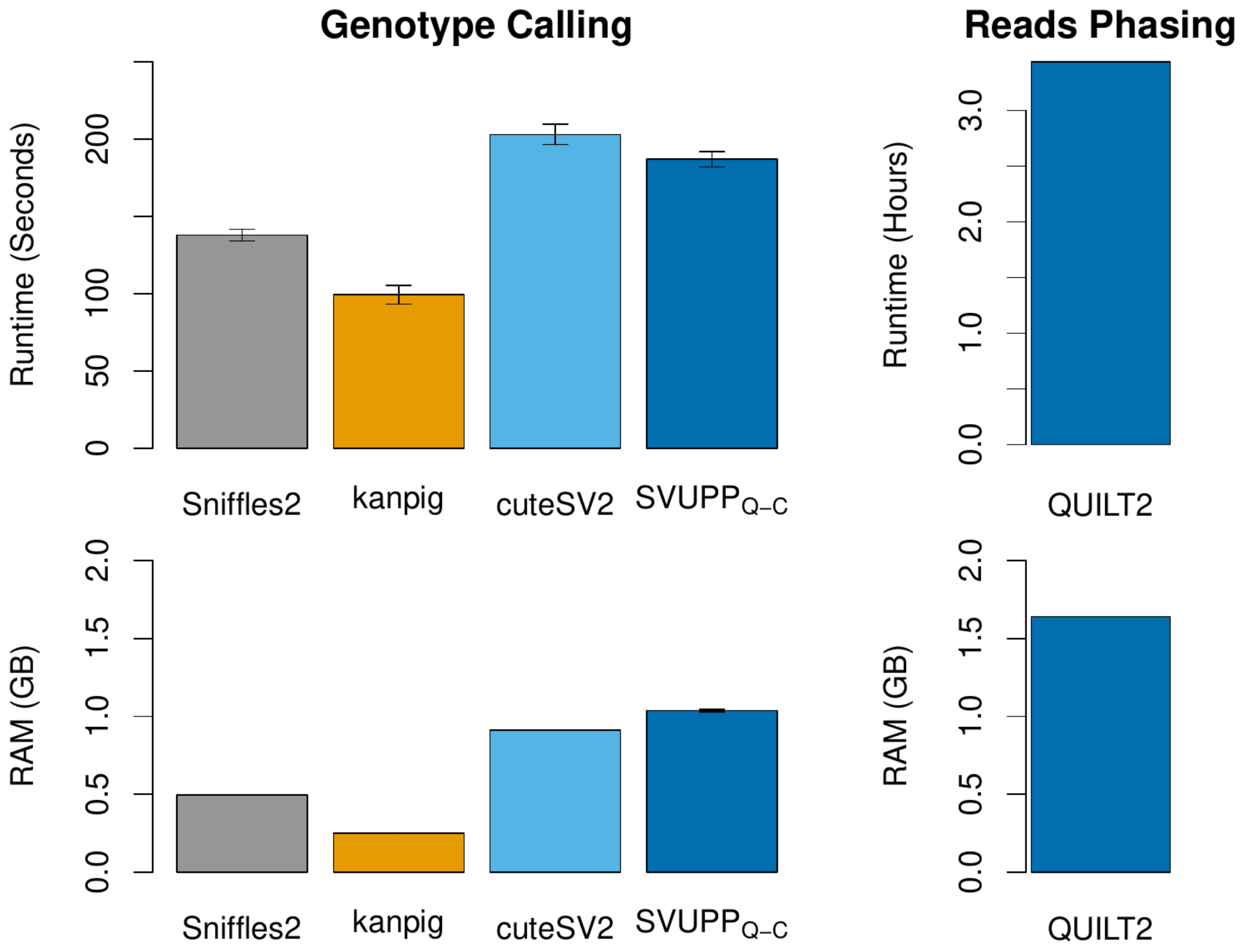

Supplementary Figure 9. Computational cost of each method. The standard error of multiple runs was shown as the error bar. *$SVUPP_{Q\text{-}C,}$* employs a customized version of cuteSV2 and takes the reads phasing information outputted by *QUILT2* to genotype SVs . *QUILT2* runs parallely and independently for each genomic chunk of average 5MB, which takes ~137 seconds per chunk on average. There are 542 chunks in total genomewide. All programs run with six threads except that *QUILT2* used one thread for each chunk. And the runtime of using six threads is shown.

Supplementary tables

Supplementary Table 1. Call rates for depth=10X force calling on platinum benchmark

**Force call rate (Depth=10X)**	**No-neighbor SVs (96278 SV genotypes)**				
	**GQ ≥ 0**	**GQ ≥ 1**	**GQ ≥ 10**	**GQ ≥ 20**	**GQ ≥ 30**
Sniffles2	99.7%	74.4%	65.1%	49.0%	29.0%
kanpig	93.3%	92.6%	83.4%	66.6%	42.6%
cuteSV2	100.0%	100.0%	81.6%	52.8%	22.6%
SVUPP$_{Q\text{-}C}$	100.0%	100.0%	91.1%	71.0%	53.3%
	**SVs with neighbors (70385 SV genotypes)**				
Sniffles2	97.9%	73.1%	60.5%	42.8%	23.9%
kanpig	91.4%	90.8%	83.5%	70.1%	47.8%
cuteSV2	100.0%	100.0%	78.1%	48.3%	20.0%
SVUPP$_{Q\text{-}C}$	100.0%	100.0%	84.5%	60.3%	40.9%

Supplementary table 1. Call rates from force-calling on the platinum pedigree benchmarking shown in figure 1B and 1C. Call rates are split into structural variants with no neighbors, 'No-neighbor SVs', and structural variants with at least one neighbor, 'SVs with neighbors'. Call rates are shown for different minimum Genotype Quality (GQ) thresholds.

Supplementary Table 2. Percentage of genotype discordance with ONT ultra long reads.

70,000 GQ-best	Depth=5X		Depth=10X		Depth=20X		Depth=30X	
	**Disc. (95%CI)**	**GQ**	**Disc. (95%CI)**	**GQ**	**Disc. (95%CI)**	**GQ**	**Disc. (95%CI)**	**GQ**
Sniffles2	16.0(15.7;16.2)	0	10.0(9.7;10.2)	2	7.1(6.9;7.3)	14	6.9(6.7;7.1)	34
kanpig	15.5(15.2;15.7)	7	11.1(10.9;11.3)	17	9.7(9.5;10.0)	37	9.5(9.3;9.7)	59
cuteSV2	15.0(14.7;15.2)	6	8.5(8.3;8.7)	12	6.5(6.3;6.7)	31	6.2(6.1;6.4)	51
$\boldsymbol{SVUPP_{Q\text{-}C}}$	9.9(9.7;10.1)	9	6.5(6.4;6.7)	17	5.3(5.1;5.4)	41	5.1(5.0;5.3)	61

Supplementary table 2. For each of 4 different read depths, genotype discordance (Disc.) and its 95% confidence interval (CI) is listed for each of the methods *Sniffles2, kanpig, cuteSV2* and $SVUPP_{Q\text{-}C}$. In addition, the genotype quality (GQ) cutoff for making 70,000 best genotypes is shown.

Supplementary Table 3. Mendelian error patterns and the simplified notation used in the figures.

Parent genotype patterns (parent1 parent2)	Trio genotype patterns (parent1 parent2 child)	
**Parent minor allele count pattern**	**Minor allele count pattern**	**Alternative allele count pattern**
00	000	000, 222
	001*	001*, 221*
	002*	002*, 220*
01	010	010, 100, 212, 122
	011	011, 101, 211, 121
	012*	012*, 102*, 210*, 120*
02	020*	020*, 200*, 202*, 022*
	021	021, 201
11	110	110, 112
	111	111
Examples		
Example: 01<br>**Parent 1**: 0/0, GT=0<br>**Parent 2**: 0/1, GT=1	Example: 011<br>**Parent 1**: 0/0, GT=0<br>**Parent 2**: 0/1, GT=1<br>**Child**: 0/1, GT=1	Example: 211<br>**Parent 1**: 1/1, GT=2<br>**Parent 2**: 0/1, GT=1<br>**Child**: 0/1, GT=1

Supplementary Table 3. The right-most column shows all possible patterns of genotypes in a trio ordered as parent 1, parent 2, and then the child. The middle column shows a simplified pattern polarized to the minor allele in the trio where the order or the parents is ignored, i.e. 010 is the same as 100. The left column is grouping the patterns according to the genotypes of the parents. The bottom row contains examples on how to read the genotype patterns in each column.
